# Extracellular matrix turnover, angiogenesis and endothelial function in acute lung injury: relationship to pulmonary dysfunction and outcome

**DOI:** 10.1186/cc11689

**Published:** 2012-11-14

**Authors:** S Sayed, N Idriss, H Sayyed

**Affiliations:** 1 Faculty of Medicine, Assuit University, Assuit, Egypt

## Background

Acute lung injury (ALI) is a syndrome with a diagnostic criteria based on hypoxemia and a classical radiological appearance, with acute respiratory distress syndrome at the severe end of the disease. Facts recommended the occurrence of rupture of the basement membranes and interstitial matrix remodeling during ALI. Matrix metalloproteinases (MMPs) participate in tissue remodeling related with pathological conditions such as ALI. We hypothesized the interrelationships between extracellular matrix (ECM) turnover as MMP-9 and indicator of angiogenesis such as angiopoietin-2 (Ang-2) as well as plasma von Willebrand factor (vWF) and their correlation with arterial partial pressure of oxygen (PaO_2_), oxygen saturation (SaO_2_) and mortality in ALI/ARDS.

## Methods

Eighty-eight mechanically ventilated patients (68 male, mean (SD) age 61 (10) years) were compared with 40 healthy controls (36 male, mean (SD) age 57 (10)). All biomarkers were measured by ELISA. Oxygenation, body temperature, leucocytes, and platelet counts were noted.

## Results

Plasma levels of all biomarkers were significantly different among ALI/ARDS subjects (*P *< 0.001) and they inversely related to PaO_2 _and SaO_2 _and positively related to mortality. Plasma levels of MMP-9 were negatively correlated with PaO_2 _and SaO_2_% in ALI/ARDS patients (*r *= - 0.75, *P *< 0.0001 and *r *= - 0.81, *P *< 0.0001) respectively. Plasma level of Ang-2 was negatively correlated with PaO_2 _and SaO_2_% in ALI/ARDS patients (*r *= - 0.68, *P *< 0.0001 and *r *= - 0.63, *P *< 0.0001) respectively. Plasma levels of vWF were negatively correlated with PaO_2 _and SaO_2_% in ALI/ARDS patients (*r *= - 0.76, *P *< 0.0001 and *r *= - 0.69, *P *< 0.001) respectively. Elevated plasma levels of all indices were interrelated at the first day of admission.

## Conclusion

The observed diversity in plasma levels of MMP-9, Ang-2 and vWF in ALI/ARDS patients (Table 1) revealed the activity and severity of the disease, shedding more light on the pathogenesis and/or presentation of ARDS.

**Table 1:** Plasma levels of all biomarkers in cohort study on admission (first day of admission)

Mean (SD)	Healthy controls (*n *= 40)	ALI/ARDS patients (*n *= 88)	*P *value
MMP-9 (ng/ml)	^a^90.23 (17.94)	582.00 (87.36)	< 0.001
Ang-2 (ng/ml)	^a^12.48 (3.26)	297.30 (52.51)	< 0.001
vWF (U/ml)	^a^124.50 (38.69)	367.40 (64.39)	< 0.001

Data presented as mean (SD). MMP-9, matrix metalloproteinase-9; Ang-2, angiopiotein-2; vWF, von Willebrand factor. *P *value by Mann-Witney test. ALI/ARDS patients were significantly different compared with healthy controls. ^a^*P *< 0.001.

